# A Case Report of Metastatic Castration-Resistant Prostate Cancer Harboring a *PTEN* Loss

**DOI:** 10.3389/fonc.2021.731002

**Published:** 2021-09-23

**Authors:** Zin W. Myint, Derek B. Allison, Carleton S. Ellis

**Affiliations:** ^1^ Department of Internal Medicine, Division of Medical Oncology, University of Kentucky, Lexington, KY, United States; ^2^ Markey Cancer Center, University of Kentucky, Lexington, KY, United States; ^3^ Department of Urology, University of Kentucky, Lexington, KY, United States; ^4^ Department of Pathology and Laboratory Medicine, University of Kentucky, Lexington, KY, United States; ^5^ Department of Pharmacy, University of Kentucky, Lexington KY, United States

**Keywords:** metastatic castration refractory prostate cancer, PI3K/AKT pathway, carboplatin, abiraterone acetate, ATM/Chk2/p53 signal pathway

## Abstract

The treatment landscape of metastatic castration-resistant prostate cancer (mCRPC) has dramatically improved over the last decade; however, patients with visceral metastases are still faced with poor outcomes. Phosphatase and tensin homolog (*PTEN*) loss is observed in 40%–60% of mCRPC patients and is also associated with a poor prognosis. Several PI3K/AKT/mTOR pathway inhibitors have been studied, with disappointing anti-tumor activity. Here, we present a case of a patient with heavily treated mCRPC who had a modest tumor response to concurrent carboplatin, abiraterone acetate/prednisone, and liver-directed radiation therapy. We discuss the potential rationale supporting the use of this combination therapy and its safety in mCRPC. While the underlying basic mechanism of our patient’s anti-tumor response remains uncertain, we suggest that further prospective studies are warranted to evaluate whether this combination therapy is effective in this population of patients with pre-treated mCRPC and *PTEN* loss.

## Introduction

Visceral metastases in men with metastatic castration-resistant prostate cancer (mCRPC) occur at a very late stage of disease. One retrospective study showed that the rate of radiologically detected visceral metastases before death from prostate cancer was 32% (1). In the vast majority of patients with visceral metastases, there are also detectable metastases at other sites such as bone and regional lymph nodes (1). The site of metastases impacts the expected survival of a patient. A prior meta-analysis showed that the poorest overall survival is seen in men with liver metastases (13.5 months) followed by lung metastases (19.4 months), non-visceral bone metastases (21.3 months), and lymph node-only metastases (31.6 months) (2).

The treatment landscape of mCRPC has dramatically improved over the last decade. The development of potent androgen synthesis and receptor inhibitors (3, 4); chemotherapy with taxanes alone or in combination with a platinum (5–8); poly (ADP-ribose) polymerase (PARP) inhibitors in patients who carry DNA homologous recombination repair gene-mutations (9); immunotherapy in men with high microsatellite instability (10); and prostate-specific membrane antigen (PSMA) targeted radiopharmaceutical agents (11) have significantly prolonged overall survival and progression-free survival in mCRPC. Still, the disease is incurable and patients with visceral metastases have a limited expected survival.

Loss of the tumor suppressor gene phosphatase and tensin homolog (*PTEN*) is identified in 15%–20% of primary prostate tumor samples. Upon progression to castrate-resistant disease, the incidence increases to 40%–60% (12). Loss of function of *PTEN* leads to an activation of the PI3K/AKT/mTOR pathway precipitating cell proliferation, growth, and survival. *PTEN* loss is associated with a poor prognosis and is an independent prognostic indicator of prostate-cancer-specific death (12). While several PI3K/AKT/mTOR pathway inhibitors have been studied in mCRPC, the majority of outcomes have been disappointing, with no significant anti-neoplastic activity (13–19). However, early-phase studies show that ipatasertib, a new oral small-molecule inhibitor of AKT (protein kinase B), has promising anti-tumor activity when combined with novel hormonal agents; the activity appears to be significantly increased in mCRPC patients with *PTEN* loss (20). A Phase III randomized study of ipatasertib plus abiraterone is currently ongoing (21). Currently, treatment of mCRPC patients with *PTEN* loss is challenging.

Here, we present the case of a patient with *PTEN* loss castration-resistant prostate cancer with liver-only metastases who failed multiple lines of treatment but demonstrated some modest response to the combination of abiraterone acetate/prednisone plus carboplatin and liver-directed radiation therapy.

## Case Presentation

A 62-year-old gentleman with a family history of prostate cancer and a personal history of stage II chronic lymphocytic leukemia (CLL) under observation (not required any treatment) and a right nephrectomy for stage I clear cell renal cell carcinoma (not required any systemic treatment) was diagnosed with a localized, high-risk prostate adenocarcinoma. His pre-surgery prostatic specific antigen (PSA) was 19.7 ng/ml, and he had a Gleason score of 4 + 5 = 9 in all prostate biopsy cores. He underwent a radical prostatectomy with bilateral pelvic lymphadenectomy in September 2016. Final pathology confirmed prostate adenocarcinoma with a Gleason score of 4 + 5 = 9 with invasion of periprostatic fat and the seminal vesicle as well as perineural invasion: pT3bN0. The pathology also revealed positive surgical margins. Post-surgery PSA was 2.39 ng/ml. He received bicalutamide and androgen deprivation therapy (ADT) with leuprolide for 6 months from his local urologist but did not receive salvage radiation. His PSA became undetectable with ADT. Germline genetic testing was performed using the Ambry CancerNext^®^ test and was negative.

**Figure 1:**
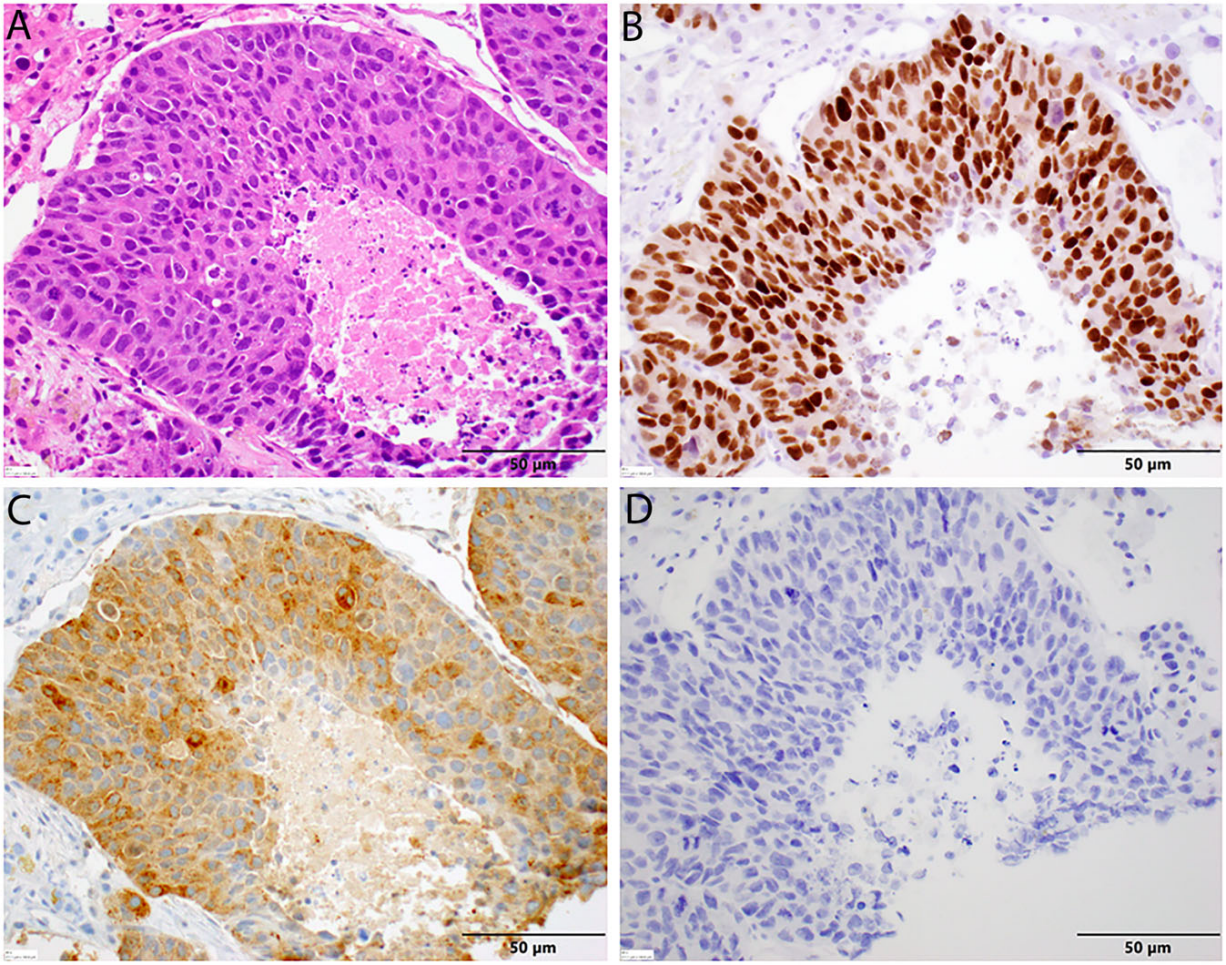
Histopathology of liver biopsy. **(A)** Metastatic prostate adenocarcinoma displaying significant nuclear enlargement and pleomorphism, prominent nucleoli, mitotic figures, and comedo-type central necrosis in this representative field. Note the absence in neuroendocrine features and the surrounding benign hepatocytes [H&E stain, 40× magnification]. **(B)** Diffuse nuclear positivity with NKX3.1 in tumor cells [NKX3.1 stain, 40× magnification]. **(C)** Diffuse cytoplasmic positivity with PSA in tumor cells. **(D)** No cytoplasmic staining with chromogranin in tumor cells [chromogranin, 40× magnification].

The patient presented to our medical oncology clinic in January 2020 with a 2-month history of lower abdominal pain, anal spasms, constipation, and significant lower urinary tract symptoms with a severe International Prostate Symptom Score (IPSS) of 33. He denied loss of appetite and his weight had been maintained. His PSA had risen to 2.3 in December 2019 in a previous record, but at our initial visit, it was elevated to 11.70 ng/ml and further elevated to 41.78 ng/ml within 4 weeks. A computerized tomography (CT) scan of his chest and abdomen and pelvis demonstrated numerous liver lesions and extensive sub-centimeter supraclavicular, mediastinal, and bilateral axillary lymphadenopathy. A nuclear bone scan was negative for bony metastases. Subsequently, a whole-body positron emission tomography/CT scan was performed to evaluate for a Richter’s transformation given his CLL history. The scan showed hypermetabolic changes in a left hepatic lesion with an standardized uptake value (SUV) of 10.2; a caudate lobe lesion with an SUV of 11; a right dome of the liver lesion with an SUV of 8.0; hypermetabolic bilateral iliac nodes with SUVs of 3.8 and 2.7; and no fluorodeoxyglucose avidity above blood pool in his supraclavicular, bilateral axillary, mediastinal, and bilateral perihilar nodes. There was no evidence of bone marrow infiltration. A liver biopsy was obtained given it demonstrated the highest uptake value and showed metastatic carcinoma that was strongly positive for the prostate-specific immunohistochemical (IHC) markers NKX3.1 and PSA and negative for the neuroendocrine markers chromogranin and synaptophysin (**Figure 1**).

**Figure 2:**
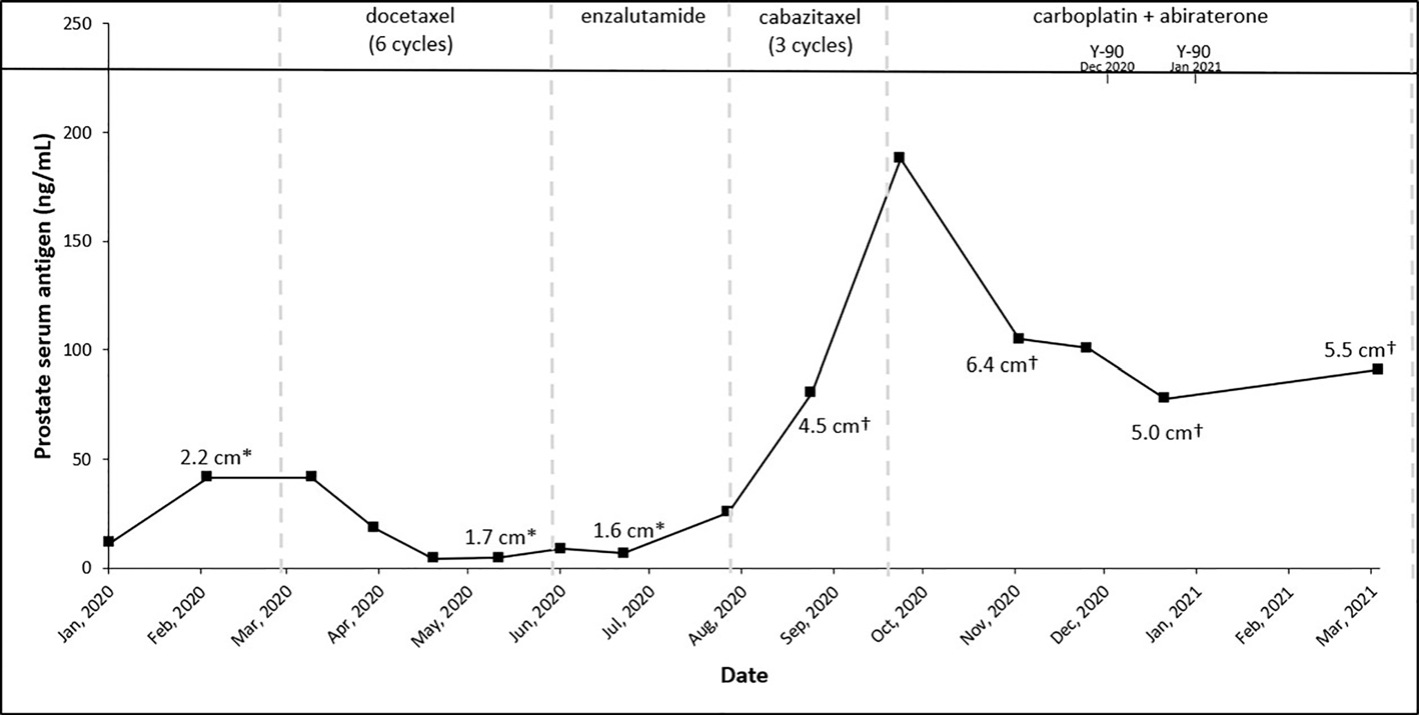
Trends of treatment, prostate serum antigen, and tumor size across patient’s treatment course. *Right inferior lobe lesion and ^†^segment 7 lesion.

**Figure 3:**
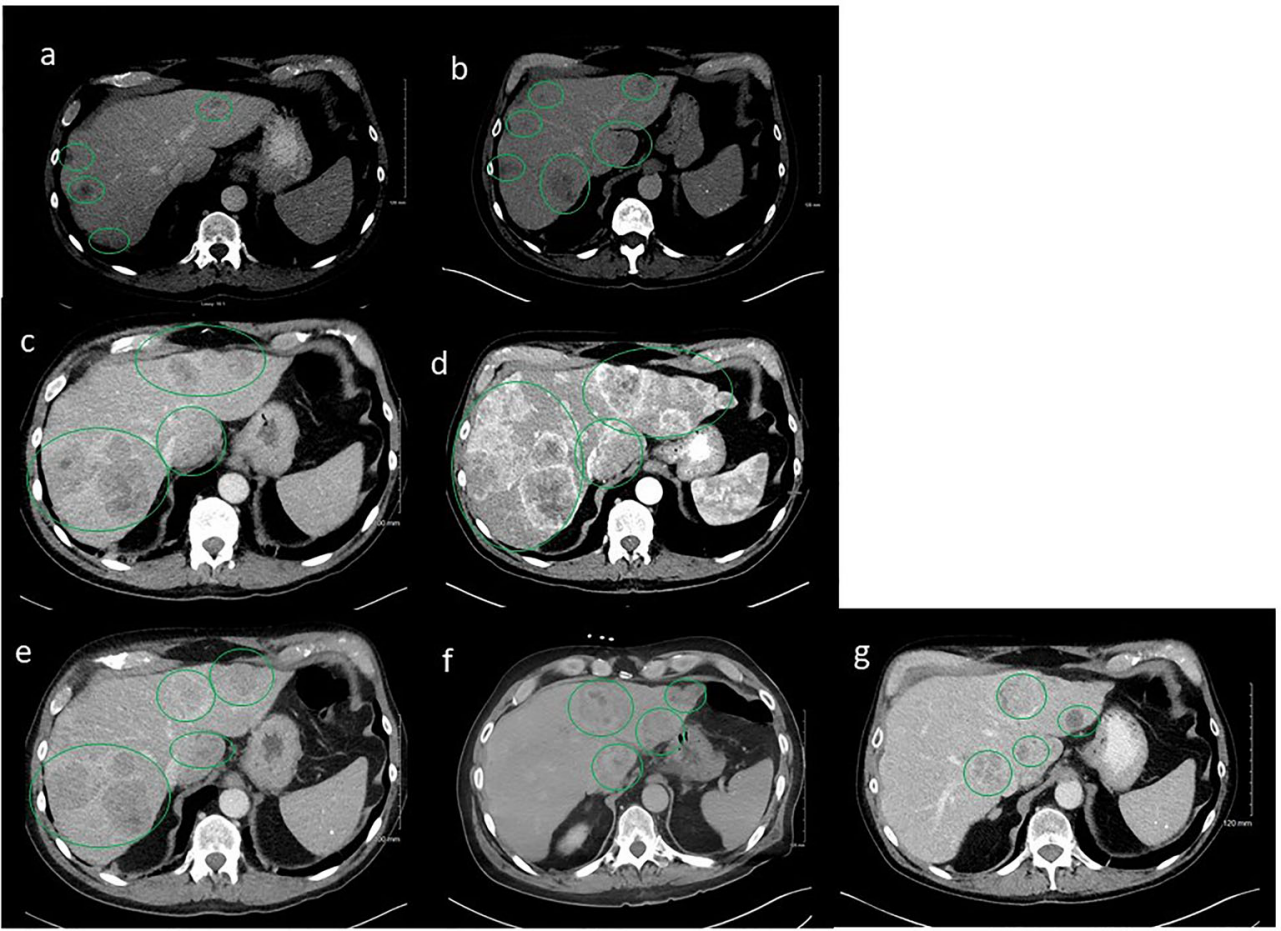
Serial computerized tomography (CT) images of abdomen and pelvis with contrast of the patient’s tumor after sequencing treatments. Green oval cycles represent tumors. **(A)** Prior to docetaxel, March 2020. **(B)** Six cycles after docetaxel, June 2020. **(C)** Two months after enzalutamide, August 2020. **(D)** After three cycles of cabazitaxel, October 2020. **(E)** After Y-90 embolization to the right lobe of liver concurrent with carboplatin/abiraterone acetate, December 2020. **(F)** After Y-90 embolization to the left lobe of liver concurrent with carboplatin/abiraterone acetate, January 2021. **(G)** On carboplatin/abiraterone acetate, May 2021.

In light of these findings, the patient was started on leuprolide 22.5 mg plus docetaxel 75 mg/m^2^. He received six cycles of docetaxel from March 2020 to June 2020 and tolerated the treatment well with no major treatment-related side effects except some mild fatigue. PSA trends shown in **Figure 2**. Repeat CT scans after three cycles of docetaxel + ADT showed stable disease; however, the scans repeated after six cycles showed disease progression in the liver (**Figures 3A, B**).

**Figure 4:**
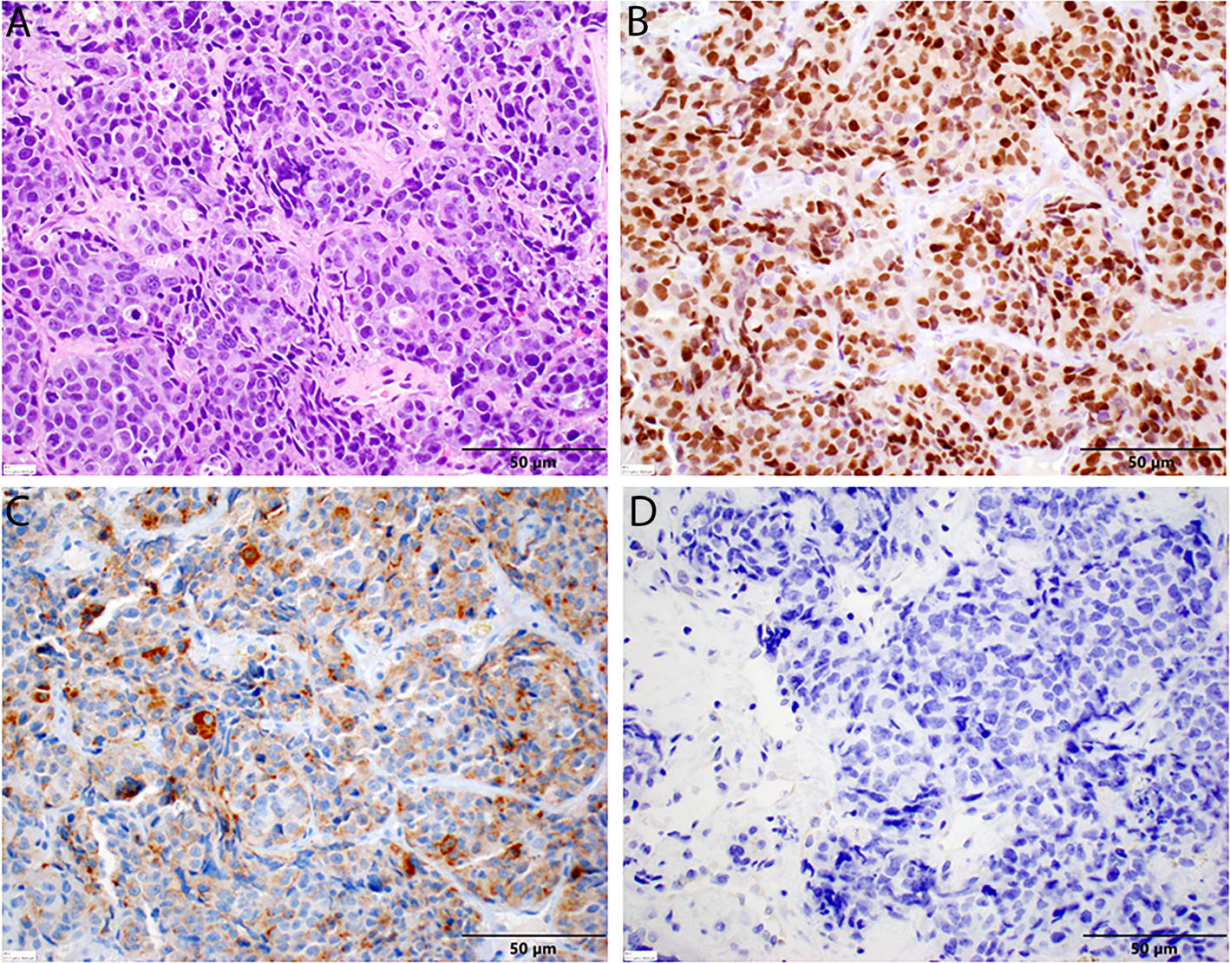
Histopathology of second liver biopsy. **(A)** Metastatic prostate adenocarcinoma displaying similar features to the previous sample, including significant nuclear enlargement and pleomorphism, prominent nucleoli, mitotic figures, and single-cell necrosis in this representative field. Again, note the absence of neuroendocrine features [H&E stain, 40× magnification]. **(B)** Diffuse nuclear positivity with NKX3.1 in tumor cells [NKX3.1 stain, 40× magnification]. **(C)** Diffuse cytoplasmic positivity with PSA in tumor cells. **(D)** No cytoplasmic staining with chromogranin in tumor cells [chromogranin, 40× magnification].

A repeat liver biopsy was again consistent with metastatic prostatic adenocarcinoma with similar morphology and IHC profile to the previous biopsy (**Figure 4**). Molecular testing through Caris Life Sciences was performed, and it showed positive for androgen receptor, *PTEN* loss in exon 2c.164, CDKN1B exon 1p.p92fs, tumor mutation burden (5 mutations/Mb), and stable microsatellite instability, and negative for NTRK1/2/3, ATM, BRCA1, BRCA2, FANCA, PALB2, RAD51C, and RAD51D. Second-line therapy with enzalutamide 160 mg was started in July 2020, but in August 2020, he presented to the emergency department with intractable right upper quadrant pain and CT scans showed progression of the hepatic masses with small, new infiltrative lesions (**Figure 3C**). PSA trends are shown in **Figure 2**. Due to the rapid progression, we switched to cabazitaxel 20 mg/m^2^ with G-CSF as third-line therapy; the patient received three cycles from September to October 2020. While on cabazitaxel, his PSA dramatically increased to 188 ng/ml and repeat scans again demonstrated worsening of his extensive hepatic metastases (**Figure 3D**). AR-V7 testing was sent and was negative. We initiated abiraterone acetate 1000 mg daily/prednisone 5 mg twice daily combined with carboplatin AUC 5 every 3 weeks in November 2020 given *PTEN* loss. He also underwent Y-90 embolization of the right lobe of his liver in December 2020 (**Figure 3E**) and a second Y-90 embolization of the left lobe of his liver in January 2021 (**Figure 3F**). He was instructed to continue his abiraterone acetate/prednisone regimen throughout his Yttrium-90 (Y-90) embolization. Y-90 embolization is a type of radiation using resin or glass microspheres containing ^90^Y administered directly into the hepatic arteries. However, the carboplatin was held 2 weeks prior and 2 weeks after Y-90 embolization. He received continuous ADT as backbone. He tolerated the treatment well without having any major side effects except grade 2 fatigue and grade 2 nausea/vomiting. His PSA slowly trended down and became stable, as shown in **Figure 2**. Repeat CT scans showed a partial response in the liver. He remains on the same chemotherapy/hormonal therapy combination at the time of writing with stable response (**Figure 3G**).

## Discussion

To our knowledge, abiraterone acetate/carboplatin/radiation combination therapy has never been studied in mCRPC patients; we report the first case on this chemotherapy/hormonal therapy/radiation therapy combination. This case demonstrates the clinical utility of the above combination therapy in patients with metastatic CRPC with *PTEN* loss.

Prostate cancer is a heterogenous disease and a small subset of the prostate adenocarcinoma population can present with aggressive clinical features like neuroendocrine origin. This subpopulation frequently carries some driver molecular alterations in retinoblastoma-associated protein 1 (*RB1*), tumor protein 53 (*TP53*), and/or *PTEN* (22). These alterations have been associated with abnormal cell proliferation and increased DNA damage response defects through activation of Akt signaling (23).

Loss of *PTEN* by mono- and biallelic deletions or mutations is among the most frequently observed molecular aberrations in localized and metastatic prostate cancer. *PTEN* loss is identified in 15%–20% of primary prostate tumor samples (12). Upon progression to castrate-resistant disease, the incidence increases to 40%–60% (12). *PTEN* loss is known to be associated with a poor prognosis (12). *PTEN* plays a crucial role as a tumor suppressor in cell cycle by controlling both G1/S and G2/M transitions (24). Loss of *PTEN* promotes activation of the PI3K/AKT/mTOR signaling pathway, which modulates several downstream pathways. This signaling pathway also causes abnormal cell proliferation and survival (25–27). *PTEN* regulates *p53* by modulating its DNA binding activity. *PTEN* and *p53* both regulate the DNA damage response pathway by promoting nucleotide excision repair (NER) following ionizing radiation damage (28). When *PTEN* function is lost, Akt signaling pathways are activated through inappropriate activation of Chk1 (29) and, thus, impairs DNA damage repair and the DNA damage response pathway (28).

Platinum compounds as monotherapy or combination therapy have shown promising activity in mCRPC (7, 8). Platinum-based agents cause mono-, inter-, or intra-strand crosslinking of DNA triggering DNA damage, which activates ATM/Chk2/p53 signaling, inducing apoptosis and cell cycle arrest (30). Interestingly, androgen receptor signaling also regulates DNA repair genes of both non-homologous end joining (NHEJ) and homologous recombination (HR) repair pathways (31). Preclinical studies (both *in vitro* and *in vivo* models) have demonstrated the synergistic combinations of radiation and novel androgen synthesis inhibitors (abiraterone acetate or enzalutamide) in both androgen-dependent and androgen-independent prostate cancer (31, 32). The rationale is that the ionizing radiation enhances DNA damage, which then activates the ATM/Chk2/p53 signaling pathway promoting cell cycle arrest (33, 34). Anti-androgen therapy further augments by decreasing DNA repair genes and, thus, inducing synthetic lethality and causing apoptosis of prostate cancer cells (31, 35). Recent early-phase studies further confirmed the clinical efficacy data of these synergistic combinations (36–38). Phase III studies are ongoing.

We hypothesize that a response was seen in this case because carboplatin and radiation both induce DNA damage through the ATM/Chk2/p53 pathway, and the loss of *PTEN* activates the PI3K/AKT pathway and causes DNA damage repair, which is further augmented by adding anti-androgen therapy. Therefore, the combination has some synergistic or additive benefits. While the underlying basic mechanism of our patient’s anti-tumor response remains uncertain, our case highlights the possible benefit and safety of combination carboplatin/abiraterone acetate/radiation in treated mCRPC and suggests that further prospective studies are warranted to evaluate whether this combination therapy is effective in this population.

## Data Availability Statement

The original contributions presented in the study are included in the article/supplementary material. Further inquiries can be directed to the corresponding author.

## Ethics Statement

Ethical review and approval was not required for the study on human participants in accordance with the local legislation and institutional requirements. Written informed consent to participate in this study was provided by the participants’ legal guardian/next of kin.

## Author Contributions

ZM and CE: drafting of the article, acquisition of data, and final approval of the manuscript. DA: providing histopathology pictures and final approval of the manuscript. All authors contributed to the article and approved the submitted version.

## Funding

This funding is supporting the University of Kentucky Markey Cancer Center’s Research Communications Office. They assisted with preparation of this manuscript.

## Conflict of Interest

The authors declare that the research was conducted in the absence of any commercial or financial relationships that could be construed as a potential conflict of interest.

## Publisher’s Note

All claims expressed in this article are solely those of the authors and do not necessarily represent those of their affiliated organizations, or those of the publisher, the editors and the reviewers. Any product that may be evaluated in this article, or claim that may be made by its manufacturer, is not guaranteed or endorsed by the publisher.
